# Complete Genome Sequence of *Phreatobacter* sp. Strain NMCR1094, a Formate-Utilizing Bacterium Isolated from a Freshwater Stream

**DOI:** 10.1128/MRA.00860-19

**Published:** 2019-09-12

**Authors:** Kiwoon Baek, Ahyoung Choi

**Affiliations:** a Bioresources Industrialization Support Department, Nakdonggang National Institute of Biological Resources (NNIBR), Sangju, Republic of Korea; b Microbial Research Department, Nakdonggang National Institute of Biological Resources (NNIBR), Sangju, Republic of Korea; Georgia Institute of Technology

## Abstract

Phreatobacter sp. strain NMCR1094 was isolated from a freshwater stream. In this study, we report the complete genome sequence of strain NMCR1094, which contains 4,974,952 bp with 65.8% G+C content and 4,701 predicted coding sequences. In particular, the *Phreatobacter* sp. NMCR1094 genome contains a formate dehydrogenase region.

## ANNOUNCEMENT

The type species Phreatobacter oligotrophus, belonging to the genus Phreatobacter of the class *Alphaproteobacteria*, was originally isolated from ultrapure water from a water storage tank (1). Presently, three species in this genus with a valid name have been published (http://www.bacterio.net/). The bacteria classified under the genus *Phreatobacter* are strictly aerobic, motile, and Gram-negative rods (1–3).

Phreatobacter sp. strain NMCR1094 (=FBCC-B2502 =KACC 19706 =NBRC 113394) was isolated from the surface of freshwater in Yeongdeok, Republic of Korea (36°24′41.3″N, 129°21′51.0″E), using a standard dilution plating method on R2A agar (BD Difco) medium. Strain NMCR1094 is a novel Gram-negative, aerobic, motile (by means of a polar flagellum), and rod-shaped bacterium. The 16S rRNA gene sequence of strain NMCR1094 was obtained from the complete genome sequence. The resulting 16S rRNA gene sequence was compared with sequences in the EzBioCloud database (4), which revealed that strain NMCR1094 was the most closely related to Phreatobacter cathodiphilus (98.7% similarity), followed by Phreatobacter stygius (98.5%), and Phreatobacter oligotrophus (98.4%). In the phylogenetic tree based on 16S rRNA gene sequences, strain NMCR1094, *P. cathodiphilus* S-12^T^, *P. stygius* YC6-17^T^, and *P. oligotrophus* PI_21^T^ formed a robust clade with high bootstrap values, indicating that strain NMCR1094 is a member of the genus *Phreatobacter*. The aim of the present study was to sequence the genome of the strain NMCR1094 in order to elucidate its metabolic potential and taxonomic position.

Strain NMCR1094 was grown aerobically at 25°C in the R2A agar medium used for the isolation of pure culture. For sequencing the complete genome, genomic DNA from strain NMCR1094 was extracted and further purified using the DNeasy blood and tissue kit (Qiagen) and Wizard genomic DNA purification kit (Promega), respectively. Sequencing was performed on the PacBio RS II platform (Pacific Biosciences, USA) using one single-molecule real-time (SMRT) cell at DNA Link (Seoul, South Korea), producing 217,315 bp of long reads and 1,851,789,035 bp after subread filtering. The whole-genome *de novo* assembly was carried out with Hierarchical Genome Assembly Process 3.0 (HGAP 3.0) (5). As the estimated genome size was 4,974,952 bp and the average coverage was 183×, after preassembly, 6,226 error-corrected long subreads (seed bases; 150,014,664 bp) were generated and *de novo* assembled for making the whole-genome sequence. As a result of the HGAP process, we obtained an *N*_50_ contig value of 4,974,952 bp and a total contig length of 4,974,952 bp, using a polishing process. The finalized genome was circularized manually using CLC Genomics Workbench v8.0 (CLC bio, USA), and putatively ambiguous areas were visually inspected.

The complete genome sequence of NMCR1094 is composed of a single circular chromosome. Putative gene-coding sequences (CDSs) from the assembled contigs were identified using Glimmer v3.02 (6), and open reading frames (ORFs) were obtained. These ORFs were searched using Blastall alignment against the NCBI nonredundant protein database (nr) for all species. The data were submitted to the Rapid Annotations using Subsystems Technology (RAST) server (7) and the National Center for Biotechnology Information (NCBI) genome sequence database. Identification of potential coding sequences was accomplished using the Basic Local Alignment Search Tool (BLAST) against the UniProt (8), Pfam (9), and Clusters of Orthologous Groups (COGs) (10) databases. Signal peptides and transmembrane helices were predicted using SignalP 4.1 (11) and TMHMM v2.0 (12). Genes for rRNA, tRNA, and other miscellaneous features were predicted using RNAmmer v1.2 (13), tRNAscan-SE v1.21 (14), and Rfam v12.0 (15). Automatic detection of clustered regularly interspaced palindromic repeats was conducted using MinCED v0.2.0 (16). Default parameters were used for all software programs unless otherwise noted. The carbohydrate-active and associated binding modules in strain NMCR1094 were determined using the Carbohydrate-Active enZyme (CAZy) database (http://www.cazy.org/) (17).

**FIG 1:**
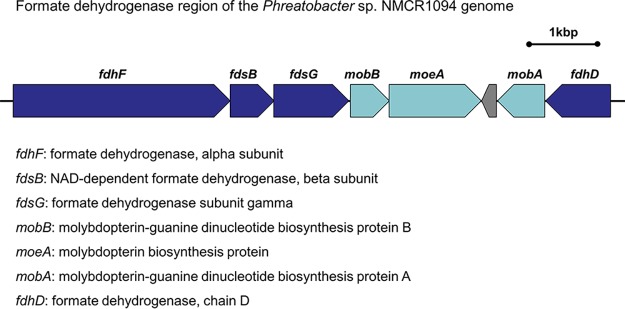
Formate dehydrogenase gene cluster of the *Phreatobacter* sp. NMCR1094 genome.

The complete genome size is 4,974,952 bp with 65.8% G+C content. Gene prediction revealed that this genome comprises 4,701 CDSs, 48 tRNA genes, and 6 rRNA genes. The genes were classified into 21 COG functional categories. According to the annotations assigned using the CAZyme database, the genome of strain NMCR1094 contained 82 carbohydrate-active enzyme genes that include 17 genes encoding glycoside hydrolases (GHs), 58 genes encoding glycosyltransferases (GTs), 5 genes encoding carbohydrate esterases (CEs), and 2 genes encoding carbohydrate-binding modules (CBMs). These substances are responsible for the potential utilization of carbohydrates. The *Phreatobacter* sp. NMCR1094 genome contains a formate dehydrogenase gene cluster (Fig. 1). The genes *fdhF* (NMCR1094_02996), *fdsB* (NMCR1094_02997), *fdsG* (NMCR1094_02998), and *fdhD* (NMCR1094_03003) are predicted to encode subunits of formate dehydrogenase (FDH), which catalyzes the final step in the pathway involved in the reversible conversion of formate to CO_2_ (18). *mobB* (NMCR1094_02999), *moeA* (NMCR1094_03000), and *mobA* (NMCR1094_03002) are predicted to encode proteins for the synthesis of a molybdenum cofactor essential for the activity of most bacterial molybdoenzymes (19). Therefore, the genomic information reveals novel insights into formate dehydrogenase in oligotrophic freshwater environments.

### Data availability.

The genome sequence of *Phreatobacter* sp. NMCR1094 has been deposited in GenBank under accession number CP039865. The associated BioProject and BioSample accession numbers are PRJNA533000 and SAMN11431406, respectively. The version described in this paper is the first version.

## References

[1] Tóth EM, Vengring A, Homonnay ZG, Kéki Z, Spröer C, Borsodi AK, Márialigeti K, Schumann P 2014 *Phreatobacter oligotrophus* gen. nov., sp. nov., an alphaproteobacterium isolated from ultrapure water of the water purification system of a power plant. Int J Syst Evol Microbiol 64:839–845. doi:10.1099/ijs.0.053843-0. 24277862

[2] Lee SD, Joung Y, Cho J-C 2017 *Phreatobacter stygius* sp. nov., isolated from pieces of wood in a lava cave and emended description of the genus *Phreatobacter*. Int J Syst Evol Microbiol 67:3296–3300. doi:10.1099/ijsem.0.002106. 28867003

[3] Kim SJ, Ahn JH, Heo J, Cho H, Weon HY, Hong SB, Kim JS, Kwon SW 2018 *Phreatobacter cathodiphilus* sp. nov., isolated from a cathode of a microbial fuel cell. Int J Syst Evol Microbiol 68:2855–2859. doi:10.1099/ijsem.0.002904. 30016224

[4] Yoon SH, Ha SM, Kwon S, Lim J, Kim Y, Seo H, Chun J 2017 Introducing EzBioCloud: a taxonomically united database of 16S rRNA and whole genome assemblies. Int J Syst Evol Microbiol 67:1613–1617. doi:10.1099/ijsem.0.001755. 28005526 PMC5563544

[5] Chin C-S, Alexander DH, Marks P, Klammer AA, Drake J, Heiner C, Clum A, Copeland A, Huddleston J, Eichler EE, Turner SW, Korlach J 2013 Nonhybrid, finished microbial genome assemblies from long-read SMRT sequencing data. Nat Methods 10:563–569. doi:10.1038/nmeth.2474. 23644548

[6] Delcher AL, Bratke KA, Powers EC, Salzberg SL 2007 Identifying bacterial genes and endosymbiont DNA with Glimmer. Bioinformatics 23:673–679. doi:10.1093/bioinformatics/btm009. 17237039 PMC2387122

[7] Aziz RK, Bartels D, Best AA, DeJongh M, Disz T, Edwards RA, Formsma K, Gerdes S, Glass EM, Kubal M, Meyer F, Olsen GJ, Olson R, Osterman AL, Overbeek RA, McNeil LK, Paarmann D, Paczian T, Parrello B, Pusch GD, Reich C, Stevens R, Vassieva O, Vonstein V, Wilke A, Zagnitko O 2008 The RAST server: Rapid Annotations using Subsystems Technology. BMC Genomics 9:75. doi:10.1186/1471-2164-9-75. 18261238 PMC2265698

[8] Wu CH, Apweiler R, Bairoch A, Natale DA, Barker WC, Boeckmann B, Ferro S, Gasteiger E, Huang H, Lopez R, Magrane M, Martin MJ, Mazumder R, O’Donovan C, Redaschi N, Suzek B 2006 The Universal Protein Resource (UniProt): an expanding universe of protein information. Nucleic Acids Res 34:D187–D191. doi:10.1093/nar/gkj161. 16381842 PMC1347523

[9] Punta M, Coggill PC, Eberhardt RY, Mistry J, Tate J, Boursnell C, Pang N, Forslund K, Ceric G, Clements J, Heger A, Holm L, Sonnhammer EL, Eddy SR, Bateman A, Finn RD 2012 The Pfam protein families database. Nucleic Acids Res 40:D290–D301. doi:10.1093/nar/gkr1065. 22127870 PMC3245129

[10] Tatusov RL, Fedorova ND, Jackson JD, Jacobs AR, Kiryutin B, Koonin EV, Krylov DM, Mazumder R, Mekhedov SL, Nikolskaya AN, Rao BS, Smirnov S, Sverdlov AV, Vasudevan S, Wolf YI, Yin JJ, Natale DA 2003 The COG database: an updated version includes eukaryotes. BMC Bioinformatics 4:41. doi:10.1186/1471-2105-4-41. 12969510 PMC222959

[11] Petersen TN, Brunak S, von Heijne G, Nielsen H 2011 SignalP 4.0: discriminating signal peptides from transmembrane regions. Nat Methods 8:785–786. doi:10.1038/nmeth.1701. 21959131

[12] Krogh A, Larsson B, Von Heijne G, Sonnhammer EL 2001 Predicting transmembrane protein topology with a hidden Markov model: application to complete genomes. J Mol Biol 305:567–580. doi:10.1006/jmbi.2000.4315. 11152613

[13] Lagesen K, Hallin P, Rødland EA, Stærfeldt HH, Rognes T, Ussery DW 2007 RNAmmer: consistent and rapid annotation of ribosomal RNA genes. Nucleic Acids Res 35:3100–3108. doi:10.1093/nar/gkm160. 17452365 PMC1888812

[14] Lowe TM, Eddy SR 1997 tRNAscan-SE: a program for improved detection of transfer RNA genes in genomic sequence. Nucleic Acids Res 25:955–964. doi:10.1093/nar/25.5.955. 9023104 PMC146525

[15] Griffiths-Jones S, Moxon S, Marshall M, Khanna A, Eddy SR, Bateman A 2005 Rfam: annotating non-coding RNAs in complete genomes. Nucleic Acids Res 33:D121–D124. doi:10.1093/nar/gki081. 15608160 PMC540035

[16] Bland C, Ramsey TL, Sabree F, Lowe M, Brown K, Kyrpides NC, Hugenholtz P 2007 CRISPR recognition tool (CRT): a tool for automatic detection of clustered regularly interspaced palindromic repeats. BMC Bioinformatics 8:209. doi:10.1186/1471-2105-8-209. 17577412 PMC1924867

[17] Lombard V, Golaconda Ramulu H, Drula E, Coutinho PM, Henrissat B 2014 The Carbohydrate-Active Enzymes database (CAZy) in 2013. Nucleic Acids Res 42:D490–D495. doi:10.1093/nar/gkt1178. 24270786 PMC3965031

[18] Ferry JG 1990 Formate dehydrogenase. FEMS Microbiol Rev 7:377–382. doi:10.1111/j.1574-6968.1990.tb04940.x. 2094290

[19] Schwarz G 2005 Molybdenum cofactor biosynthesis and deficiency. Cell Mol Life Sci 62:2792–2810. doi:10.1007/s00018-005-5269-y. 16261263 PMC11145942

